# Synergistic Antiviral Activity of European Black Elderberry Fruit Extract and Quinine Against SARS-CoV-2 and Influenza A Virusa

**DOI:** 10.3390/nu17071205

**Published:** 2025-03-29

**Authors:** Christian Setz, Pia Rauch, Melanie Setz, Stephan Breitenberger, Stephan Plattner, Ulrich Schubert

**Affiliations:** 1 Institute of Virology, Friedrich-Alexander University Erlangen-Nürnberg (FAU), 91054 Erlangen, Germany; christian.setz@uk-erlangen.de (C.S.); pia.rauch@uk-erlangen.de (P.R.); melanie.setz@uk-erlangen.de (M.S.); 2 Iprona Lana SpA, Industriestraße 1/6, I-39011 Lana, Italy; stephan.breitenberger@iprona.com (S.B.); stephan.plattner@iprona.com (S.P.)

**Keywords:** European black elderberry fruit extract, quinine, natural substance, anthocyanins, phenolic compounds, SARS-CoV-2, Influenza A virus, antiviral, pandemic preparedness, broad antiviral activity

## Abstract

Background/Objectives: The persistent threat of emerging respiratory RNA viruses like SARS-CoV-2 and Influenza A virus (IAV) necessitates the continuous development of effective, safe, broadly acting, and generally accessible antiviral agents. Current treatments often face limitations such as early administration requirements, resistance development, and limited global access. Natural products, like European black elderberry (*Sambucus nigra* L.; *S. nigra*) fruit extract and quinine, have been used historically against viral infections. In this study, we investigated the antiviral efficacy of a standardized black elderberry fruit extract containing 3.2% anthocyanins (EC 3.2) and, as a second natural antiviral product, quinine, against IAV and SARS-CoV-2 in vitro. Methods: Madin–Darby Canine Kidney II (MDCKII) cells were infected with IAV PR-8, while human Calu-3 lung epithelial cells were infected with Wuhan-type SARS-CoV-2. Cells were treated with varying concentrations of EC 3.2 and quinine either as mono- or combinational therapy. Viral replication was assessed using quantitative RT-PCR, and cell viability was evaluated using WST-1 assays. Results: Our results demonstrate, for the first time, that both EC 3.2 and quinine individually inhibited IAV replication in a dose-dependent manner, with IC_50_ values of approximately 1:400 for EC 3.2 and 250 nM for quinine. Most importantly, the combinational treatment exhibited a strong synergistic antiviral effect, as confirmed by the Bliss independence model (synergy scores of 14.7 for IAV, and 27.8 for SARS-CoV-2), without affecting cell viability. Conclusions: These findings suggest that the combined use of black elderberry extract and quinine might serve as an effective antiviral strategy against IAV and SARS-CoV-2, particularly since the synergistic effect allows for lower doses of each product while retaining therapeutic efficacy. In summary, this combinational in vitro approach, when expanded to other respiratory RNA viruses and confirmed in clinical studies, has the potential to open a promising avenue for pandemic preparedness.

## 1. Introduction

Even after its transition to an endemic spread, SARS-CoV-2 remains a public health menace. This is particularly true with regard to emerging variants of concern (VoCs) and the necessity for pandemic preparedness to encounter newly emerging and/or reemerging viruses in the future [1]. Several vaccines and monoclonal antibodies have been developed and globally approved. These measures could moderate the course of infection, although sterile immunity, which would prevent SARS-CoV-2 virus transmission and, thus, progressive adaptation of the pathogen, could not be achieved [2,3,4]. In addition, the protease inhibitor Paxlovid^®^ as well as the polymerase inhibitors Molnupiravir and Remdesivir have been available for antiviral treatment [5]. However, these drugs are required to be administered in the initial stages of infection. Operationally and prophylactically acting substances with subtle adverse effects have still not been made available. For these reasons, and with respect to potential future pandemic events, there is still an unmet medical need for broadly acting antiviral drugs against SARS-CoV-2 VoCs as well as other respiratory RNA viruses. At best, these drugs should be safe, readily available, and inexpensive.

Similar to the situation with SARS-CoV-2, Influenza A is a highly contagious respiratory virus causing seasonal epidemics with significant morbidity and mortality worldwide. Every year, about 10% of the world’s population become infected with Influenza, and each year about 500,000 people die due to infections [6]. Characterized by symptoms such as fever, cough, sore throat, muscle aches, and fatigue, it poses a recurring public health challenge [7]. Current prevention and treatment options include annual vaccinations, antiviral medications, like the neuraminidase inhibitors Oseltamivir, Zanamivir, and Peramivir, and symptomatic care [6]. However, issues such as antiviral resistance, variable vaccine efficacy, and limited global access to treatments highlight the ongoing medical need for improved vaccines, new effective antiviral drugs, rapid diagnostics, and equitable healthcare strategies [8]. Altogether, pandemic preparedness against upcoming emerging highly pathogenic IAVs with pandemic potential is inevitable.

Natural substances represent broadly acting antivirals and exhibit immense potential to improve our pandemic preparedness [9]. Additionally, for the reason of their natural origin, they have fewer side effects than synthetically intended drugs [9,10,11].

European black elderberry (*Sambucus nigra* L.; *S. nigra*) has been used for centuries in traditional medicine to treat cold diseases mostly due to respiratory viruses. Recent in vivo studies, conducted in animal and humans, have demonstrated that extracts from the berries of black elderberry possess antiviral properties. Moreover, they can shorten the duration and severity of upper respiratory infections in humans [12,13,14,15]. Very recently, the strong antiviral activity of black elderberry fruit extract, standardized to anthocyanins and phenolic compounds, against SARS-CoV-2 and its variants was demonstrated in vitro, suggesting its potential as an effective treatment option for COVID-19 [16].

Moreover, crude ethanol extract of *S. nigra* has shown significant antiviral properties against Infectious Bronchitis Virus (IBV), reducing viral titers by four to six orders of magnitude. The extract works by compromising viral envelopes and inducing membrane vesicles, rendering the virus non-infectious early in the infection process [17]. In addition, cyanidin-3-sambubiocide, a main anthocyanin of black elderberry extract, has been shown to be a potent inhibitor of Influenza neuraminidase. The anthocyanin interacts specifically with the active center of the neuraminidase and thereby inhibits virus release [18].

Roschek et al. identified anti-viral components in *S. nigra* extract, which inhibited human Influenza A Virus (H_1_N_1_) in vitro. Key flavonoids, including 5,7,3′,4′-tetra-O-methylquercetin, demonstrated potent anti-H_1_N_1_ activity, comparable to the neuraminidase inhibitors Oseltamivir and Amantadine [19].

Quinine can be extracted from the bark of the Cinchona tree and was used worldwide for centuries to treat feverish infections, especially malaria [20,21,22]. It served as a template for the synthesis of Hydroxy-Chloroquine (H-CQN) in 1946, which was then mainly used for the treatment of malaria. Until now, quinine is still a treatment option for severe and H-CQN-resistant cases of *malaria tropica* [21,22,23]. In addition, quinine is approved for the treatment of calf cramps and is widely used as an aromatic agent in beverages like tonic water, with its use subjected to regulatory limits.

Recently, we have shown for the first time that quinine efficiently inhibits the replication of SARS-CoV-2 in vitro [20]. Moreover, antiviral activity of quinine against Human Immunodeficiency Virus, Zika Virus, Herpes Simplex Virus, Ebola, and Dengue virus was described [21,24,25,26,27]. Interestingly, in 1946, Seeler et al. reported first enigmatic hints that quinine exhibits a benefit on Influenza infections in mice [28].

The aims of this study were (i) to evaluate the antiviral effect of *S. nigra* or quinine on the replication of IAV and (ii) to analyze their potential synergistic activity on the replication of IAV and SARS-CoV-2 in vitro.

## 2. Materials and Methods

### 2.1. Inhibitors

Liquid European black elderberry (*S. nigra*) fruit extract, branded as ElderCraft^®^ and designated EC 3.2, was provided by Iprona Lana SpA (Lana, Italy). EC 3.2 is a water-based liquid extract standardized to contain a minimum of 3.2% anthocyanins. Dry European black elderberry extract, standardized to 15% anthocyanins and designated EC 15, was also supplied by Iprona Lana SpA. Additionally, a combination product containing anthocyanins and quinine, designated as ElderCraft^®^ Q-Complex, was obtained from the same supplier. The exact compositions of EC 3.2 and EC 15 are provided in Supplementary Table S1. Total monomeric anthocyanins in ElderCraft^®^ black elderberry extract were quantified using the pH differential method based on UV–Vis spectrophotometry. This method exploits pH-dependent structural changes in anthocyanins, leading to absorbance differences [29]. A 1:4 dilution was prepared by homogenizing 5 g of *S. nigra* extract in 20 g of water, followed by further dilution with water to achieve a theoretical absorbance of 12.5 absorbance units at 520 nm. Cloudy solutions were filtered (0.45 µM CA membrane). *S. nigra* extract was then mixed with pH 1.0 (KCl) and pH 4.5 (phosphate–citrate) buffers, followed by the recording of absorbance at 520 nm using a UV–Vis spectrophotometer. As a standard, cyanidin chloride (5.3 mg, 96.9% purity) was dissolved in solution to a final volume of 20 mL. Standard solutions (2.00–13.00 mg/L) were prepared. Anthocyanin content was calculated as cyanidin-3-glucoside equivalents. The anthocyanin stability in EC 3.2 was measured for eleven months by monthly total anthocyanin quantifcation using the pH differential method (Supplementary Figure S1). Quinine was obtained as quinine sulfate from Sigma-Aldrich (St. Louis, MO, USA) and dissolved in DMSO.

### 2.2. Viruses

The “Wuhan type” virus SARS-CoV-2_PR-1_, isolated from a 61-year-old patient, was amplified in Vero B4 cells, as described previously [20]. Viral titers were determined via an endpoint titration assay. For the generation of new virus stock, virus containing cell culture supernatant was harvested 72 h post infection (hpi) and passed through a 0.45 μm pore-size filter. All virus stocks were stored at −80 °C until further usage.

The Influenza A virus isolate (IAV) A/Puerto Rico/8/34 (PR8) [30] was provided by Dr. Matthias Tenbusch (Institute of Clinical and Molecular Virology, Erlangen, Germany). Viral titers were determined via a plaque assay. For the generation of new virus stock, virus containing cell culture supernatant was harvested 48 hpi and passed through a 0.45 μm pore-size filter. All virus stocks were stored at −80 °C until further usage.

### 2.3. Infection Experiments

Calu-3 cells, exemplifying the most comprehensively studied surrogate lung cell infection model that expresses ACE2 and TMPRSS2 endogenously [31], were inoculated with SARS-CoV-2_PR-1_ (Wuhan type) with a multiplicity of infection (MOI) of 2 × 10^−2^ for 1 h, washed, and further treated with interventions. Seventy-two hours post infection, virus-containing cell culture supernatants were incubated for 10 min at 95 °C and finally used for qRT-PCR analysis. For titer determination of SARS-CoV-2 virus stocks, Calu-3 cells were infected with serial dilutions of the virus stock over 72 h. Afterwards, cells were fixed (4% PFA), permeabilized (0.5% Triton/PBS), blocked (1% BSA/PBS-T), and finally stained with a SARS-CoV-2 NP antibody (Biozol, Eching, Germany). Each endpoint of virus infection was analyzed via fluorescence microscopy and the viral titer was calculated via the method of Reed and Muench [32].

Madin–Darby Canine Kidney (MDCKII) cells [33], the standard cell culture model for studying IAV replication in vitro, were inoculated with IAV_PR8_ (MOI: 0.01) for 30 min, washed, and further treated with the indicated interventions. Forty-eight hours post infection, virus-containing cell culture supernatants were incubated for 10 min at 95 °C and finally used for qRT-PCR analysis. For titer determination of IAV_PR-8_ virus stocks, MDCKII cells were infected with serial dilutions of the virus stock for 3 h. Afterwards, cells were overlayed with 1% purified agar (Oxoid, Wesel, Germany) and incubated at 37 °C for 4 days. Each endpoint of virus infection was analyzed by counting plaque forming units (PFU), visualized following incubation of the cells with MTT 3-(4,5-Dimethylthiazol-2-yl) for 3 h. The viral titer was calculated via the method of Reed and Muench [32].

### 2.4. Cell Culture

Calu-3 cells were maintained in Minimal Essential Medium (MEM) containing 20% (*v*/*v*) inactivated fetal calf serum (FCS), 1 mM l-glutamine, 100 U/mL penicillin, 100 μg/mL streptomycin, and 1 mM sodium pyruvate.

MDCKII cells were maintained in Dulbecco’s Minimal Essential Medium (DMEM) containing 10% (*v*/*v*) inactivated FCS, 1 mM l-glutamine, 100 U/mL penicillin, and 100 μg/mL streptomycin.

### 2.5. Assessment of Cell Viability

The viability of uninfected but treated cells was assessed using the water-soluble tetrazolium salt (WST)-1 assay (Cat.: 5015944001, Roche, Penzberg, Germany), according to the manufacturer’s instructions.

### 2.6. Determination of the Amount of Viral RNA Copies from Released Viruses via qRT-PCR

The amount of viral SARS-CoV-2 RNA copies in the virus-containing samples was quantified using the real-time PCR Luna Universal Probe One-Step RT-PCR Kit from New England Biolabs (Cat: E3006L, Ipswich, MA, USA). This kit allows the reverse transcription, cDNA synthesis, and PCR amplification in a single step. Samples were analyzed by 7500 software v2.3 (Applied Biosystems, Waltham, MA, USA). PCR primers were designed and used as described previously in [34]. Thereby, the polynucleotide sequence contained parts of the SARS-CoV-2 Envelope (E) and RNA-dependent RNA-polymerase (RdRp) genes and was used as the standard for the determination of viral RNA copies in the experiments. The sequences of the used primers were RdRp_forward (fwd) 5′-GTG-ARA-TGG-TCA-TGT-GTG-GCG-G-3′ and RdRp_reverse (rev) 5′-CAR-ATG-TTA-AAS-ACA-CTA-TTA-GCA-TA-C-3′. The probe was 5′-CAG-GTG-GAA-/ZEN/CCT-CAT-CAG-GAG-ATG-C-3′ (Label: FAM/IBFQ Iowa Black FQ). A dsDNA-polynucleotide sequence (Integrated DNA Technologies, Coralville, IA, USA) was used as a positive control: 5′-TAA-TAC-GAC-TCA-CTA-TAG-GGT-ATT-GAG-TGA-AAT-GGT-CAT-GTG-TGG-CGG-TTC-ACT-ATA-TGT-TAA-ACC-AGG-TGG-AAC-CTC-ATC-AGG-AGA-TGC-CAC-AAC-TGC-TTA-TGC-TAA-TAG-TGT-TTT-TAA-CAT-TTG-GAA-GAG-ACA-GGT-ACG-TTA-ATA-GTT-AAT-AGC-GTA-CTT-CTT-TTT-CTT-GCT-TTC-GTG-GTA-TTC-TTG-CTA-GTT-ACA-CTA-GCC-ATC-CTT-ACT-GCG-CTT-CGA-TTG-TGT-GCG-TAC-TGC-TGC-AAT-ATT-GTT-3′. Generating a series of dilutions (10^4^, 10^5^, 10^6^, and 10^7^ copies/mL) of this standard, the experiments were quantified using a standard curve to obtain absolute values of RNA copies in the sample.

The amount of viral IAV RNA copies in the virus-containing samples was quantified using the real-time PCR GoTaq^®^ Probe qPCR one step Kit from Promega (Cat: A6120, Madison, WI, USA). This kit allows the reverse transcription, cDNA synthesis, and PCR amplification in a single step. Samples were analyzed by 7500 software v2.3 (Applied Biosystems, Waltham, MA, USA). PCR primers (Integrated DNA Technologies, Coralville, IA, USA) contained parts of the IAV Matrix gene. The sequences of the used primers were 5′Inf-A-M: 5′-AGA TGA GTC TTC TAA CCG AGG TCG-3′, 3′Inf-A-M: 5′-TGC AAA AAC ATC TTC AAG TCT CTG-3′, and 3′Inf-A-SW-M: 5′-TGC AAA GAC ATC TTC CAG TCT CTG-3′.

### 2.7. Software and Statistics

GraphPad Prism 9.0 was used for statistical analyses and to generate graphs. 7500 software v2.3 was used to evaluate the results obtained via qRT-PCR. To determine the combinatory effects of the treatment with EC 3.2. and quinine, the open-source and free web application SynergyFinder (https://synergyfinder.fimm.fi/; accessed on 12 August 2024) was used [35], and the drug interactions were analyzed via the commonly used Bliss independence model [36]. Using this model, independent effects of different small molecules assumed by stochastic processes could be analyzed [35,36]. Thereby, a Bliss synergy score >10 indicates synergistic activity, −10 to 10 represents additive effects, and <−10 suggests antagonism [35].

## 3. Results

### 3.1. European Black Elderberry Fruit Extract and Quinine Exhibit Antiviral Activity Against Influenza A Virus in MDCKII Cells

**Figure 1:**
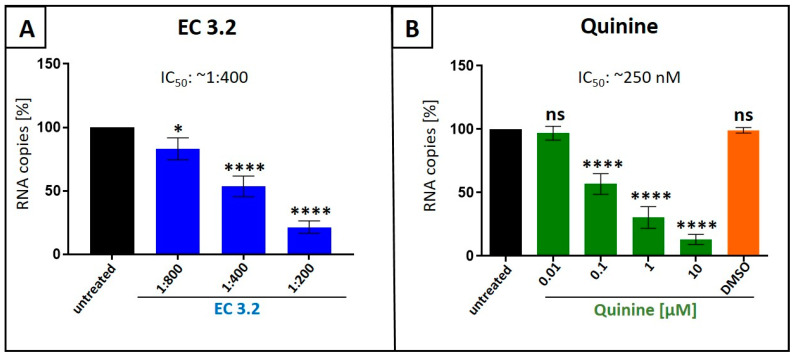
Liquid European black elderberry (EC 3.2) extract (**A**) and quinine (**B**) inhibit the replication of Influenza A virus (IAV) in MDCKII cells. MDCKII cells were infected with the IAV isolate A/Puerto Rico/8/34 (PR8) at an MOI of 0.01. Thirty minutes after infection and removal of input virus, cells were treated with indicated dilution steps of EC 3.2 (**A**) or concentrations of quinine sulfate (**B**). DMSO as a solvent control was tested at the same concentration as used in the highest concentration of quinine (10 µM). Cell culture supernatants were harvested at 2 days post infection (dpi). The virions were purified and analyzed via qRT-PCR. Bars show mean values of three independent experiments ± standard deviation. Statistical analysis was performed using a multiple comparison Kruskal–Wallis test (Anova) followed by Dunn’s post hoc test (* *p* < 0.00312; **** *p* < 0.0001; and ns = not significant versus the untreated control).

According to our current knowledge, there are no reports available showing antiviral activity of black elderberry fruit extract, or quinine, against IAV in vitro. Thus, we first analyzed if both natural compounds exhibit antiviral activity against IAV: (i) quinine, and (ii) liquid European black elderberry fruit extract from *S. nigra* (EC 3.2), a water-based liquid extract standardized to contain a minimum of 3.2% anthocyanins. Therefore, Madin–Darby Canine Kidney (MDCKII) cells were infected with the IAV isolate A/Puerto Rico/8/34 (PR8) (Figure 1). Thirty minutes post infection, serial dilutions of EC 3.2 or various concentrations of quinine were added to the cell cultures and incubated continuously. Two days post infection (dpi), cell culture supernatants were harvested, and viral production was analyzed via quantitative RT-PCR (qRT-PCR) (Figure 1).

Treatment with EC 3.2 or quinine led to a dose-dependent inhibition of IAV replication, reducing the production of progeny virions by 80% at a dilution of 1:200 EC 3.2, and by 90% with a concentration of 10 µM quinine. DMSO, a solvent control for quinine, had no influence on the replication of IAV (Figure 1B).

**Figure 2:**
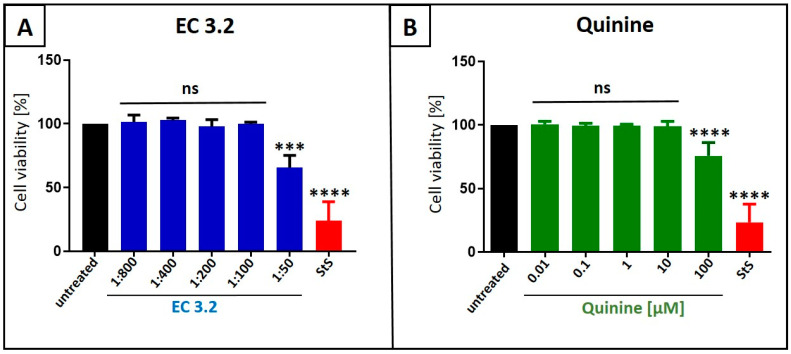
Influence of EC 3.2 (**A**) or quinine (**B**) on the cell viability of MDCKII cells. Following treatment with different dilutions of EC 3.2 or concentrations of quinine sulfate (indicated at the *x*-axis) for two days, the influence on cell viability was measured via water-soluble tetrazolium salt (WST)-1 assay. Bars represent means of three independent experiments ± SD. Staurosporine (StS, 1 µM) was used as a positive control. Statistical analysis was performed using a multiple comparison Kruskal–Wallis test (Anova) followed by Dunn’s post hoc test (*** *p* < 0.06; **** *p* < 0.0001; and ns = not significant versus the untreated control).

Water-soluble tetrazolium salt (WST)-1 assays were conducted in uninfected MDCKII cells under otherwise identical conditions as for the virus infection experiments, in order to control for potential unspecific effects of EC 3.2 or quinine treatment on cell viability. Neither EC 3.2 at dilutions up to 1:100 nor quinine at up to 100 µM, both at concentrations fully blocking IAV_PR8_ replication, affected cell viability (Figure 2). Staurosporine (1 µM) served as a positive control.

### 3.2. Combination Treatment with Black Elderberry Fruit Extract and Quinine Exhibits Synergistic Antiviral Activity Against IAV

**Figure 3:**
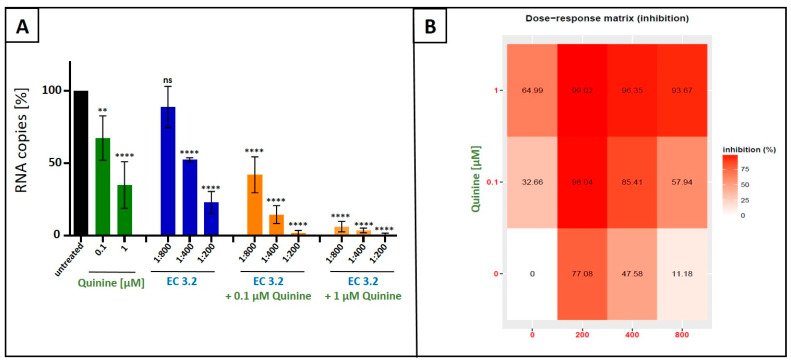
Antiviral activity of the combinatory treatment of quinine with EC 3.2 following infection with IAV_PR-8_. (**A**): MDCKII cells were infected with IAV_PR-8_ at an MOI of 0.01. Thirty minutes after infection and removal of input virus, cells were treated with indicated concentrations of quinine (green), dilution steps of EC 3.2 (blue), or the combinatory treatment of quinine and EC 3.2 (orange). Cell culture supernatants were harvested at 2 dpi. The virions were purified and analyzed via qRT-PCR. Bars show mean values of three independent experiments ± standard deviation. Statistical analysis was performed using a multiple comparison Kruskal–Wallis test (Anova) followed by Dunn’s post hoc test (** *p* < 0.01; **** *p* < 0.0001; and ns = not significant versus the untreated control). (**B**): Percentages of inhibition of viral replication following combined treatment with quinine and EC 3.2 and infection with IAV_PR-8_. The tables were created using the open-source and free web application SynergyFinder [35].

Next, we analyzed whether or not combinational treatment with EC 3.2 and quinine has additive or even synergistic antiviral activity against IAV_PR-8_. MDCKII cells were infected with IAV_PR-8_ and, 30 min post infection, different dilutions of EC 3.2 or concentrations of quinine alone or in combination were added to the cell cultures (Figure 3). Two dpi, cell culture supernatants were harvested, and viral production was analyzed via qRT-PCR (Figure 3).

Following treatment with increasing amounts of EC 3.2 (1:800–1:200) in combination with 100 nM of quinine (IC_50_ value for inhibition of IAV replication as monotherapy (Figure 1)), significant and dose-dependent reduction in replication capacity was detected, ranging from 58–98%, from the lowest to highest concentrations of EC 3.2. Treatment with the identical concentration series of EC 3.2 in the presence of 1 µM quinine resulted in even higher antiviral efficacy, leading to a reduction in viral replication ranging from 93–99%, from the lowest to highest concentrations of EC 3.2 (Figure 3A,B).

In contrast, individual treatment with EC 3.2 or quinine alone had only minor effects on viral replication at concentrations tested (Figure 3A). Single treatment with the lowest tested dilution of EC 3.2 (1:800) showed no significant influence on viral replication (Figure 3A). In contrast, the highest concentration of quinine (1 µM) reduced viral replication by max. 64% (Figure 3B). However, the combination treatment (1:800 EC 3.2 and 1 µM quinine) almost completely inhibited viral replication, which points towards a synergistic antiviral effect.

**Figure 4:**
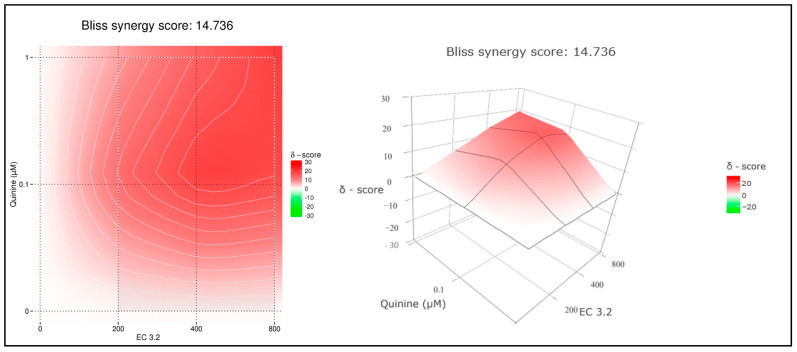
Interaction profile of quinine and EC 3.2 for determining the synergy in inhibiting the replication capacity of IAV_PR-8_. Drug interactions were analyzed using the reference model Bliss independence. The illustrations were created using the open-source and free web application SynergyFinder. The synergy calculations were performed on data derived from the experiments in MDCKII cells. The data represent means of three independent experiments. A color-coded interaction graphic was used to illustrate the Bliss synergy scores. High synergy scores are colored in red.

To quantify the effects of combination treatment, synergy scoring was conducted using the Bliss independence model. The conducted small molecule interaction analysis resulted in a Bliss synergy score of 14.7 for the combination of EC 3.2 and quinine for inhibiting replication of IAV_PR-8_ (Figure 4).

**Figure 5:**
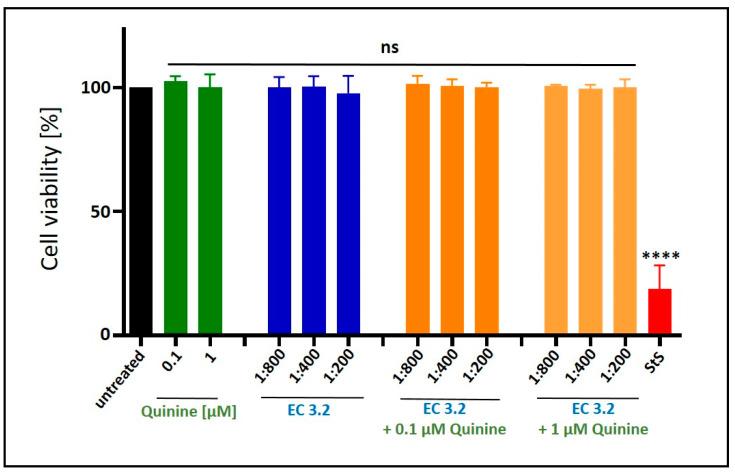
Influence of the combinatory treatment of quinine with EC 3.2 on the cell viability of MDCKII cells. Following treatment with different dilutions of EC 3.2 or concentrations of quinine sulfate (indicated at the *x*-axis) for two days, the influence on cell viability was measured via water-soluble tetrazolium salt (WST)-1 assay. Bars represent means of three independent experiments ± SD. Staurosporine (StS, 1 µM) was used as a positive control. Statistical analysis was performed using a multiple comparison Kruskal–Wallis test (Anova) followed by Dunn’s post hoc test (**** *p* < 0.0001 and ns = not significant versus the untreated control).

Water-soluble tetrazolium salt (WST)-1 assays were conducted in uninfected MDCKII cells under otherwise identical conditions as for the virus infection experiments, in order to control for potential unspecific effects of EC 3.2 or quinine treatment on cell viability. Thereby, it was demonstrated that the combinational treatment with EC 3.2 and quinine had no impact on cell viability in all tested dilutions or concentrations (Figure 5).

In summary, the results clearly show that EC 3.2 and quinine exhibit a synergistic effect to inhibit the replication of IAV. If approved in vivo, these data suggest that the combination of these two natural substances might exhibit substantial efficacy when administered in combination at relatively low doses.

### 3.3. Treatment with a Combination of European Black Elderberry Fruit Extract and Quinine Exhibits Synergistic Antiviral Activity Against SARS-CoV-2

**Figure 6:**
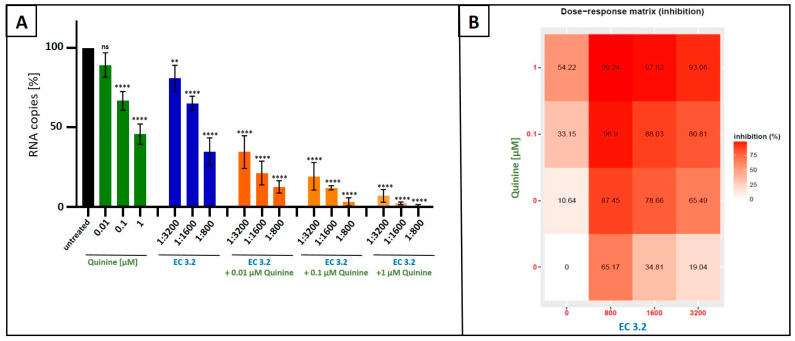
Antiviral activity of the combinatory treatment of quinine with EC 3.2 following infection with Wuhan-type SARS-CoV-2. (**A**): Calu-3 cells were infected with the clinical isolate SARS-CoV-2_PR-1_ at an MOI of 2 × 10^−2^. One hour after infection and removal of input virus, cells were treated with indicated concentrations of quinine (green), dilution steps of EC 3.2 (blue), or the combinatory treatment of quinine and EC 3.2 (orange). Cell culture supernatants were harvested at 3 dpi. The virions were purified and analyzed via qRT-PCR. Bars show mean values of three independent experiments ± standard deviation. Statistical analysis was performed using a multiple comparison Kruskal–Wallis test (Anova) followed by Dunn’s post hoc test (** *p* < 0.057; **** *p* < 0.0001; and ns = not significant versus the untreated control). (**B**): Percentages of inhibition of viral replication following combined treatment with quinine and EC 3.2 and infection with IAV_PR-8_. The tables were created using the open-source and free web application SynergyFinder [35].

Next, we wanted to analyze if the combinational treatment with EC 3.2 and quinine, as shown for IAV (Figure 3 and Figure 4), also exhibits an additive or even synergistic antiviral activity against SARS-CoV-2. Therefore, Calu-3 cells were infected with Wuhan-type SARS-CoV-2. Various dilutions of EC 3.2 or concentrations of quinine alone, or in combination, were added to the cell cultures one hour post infection (Figure 6). Two dpi, cell culture supernatants were harvested, and viral production was analyzed via qRT-PCR (Figure 6).

Treatment with increasing amounts of EC 3.2 (1:3200–1:800) in combination with 1 µM of quinine (IC_50_ value for inhibition of SARS-CoV-2 replication) results in a complete blockage of the replication capacity, independent from the dilution of EC 3.2. Treatment with the identical dilution series of EC 3.2 in the presence of 100 nM quinine also resulted in significant antiviral efficacy. Viral replication was reduced by 80–97%, from the lowest to highest concentrations of EC 3.2 (Figure 6A,B).

By contrast, treatment with EC 3.2 (1:3200) alone reduced replication by only 19%, and 10 nM quinine alone had no significant effect. (Figure 6A). However, combination treatment (1:3200 EC 3.2 and 10 nM quinine) inhibited viral replication by 65–87%, which again clearly points towards a strong synergistic antiviral effect.

**Figure 7:**
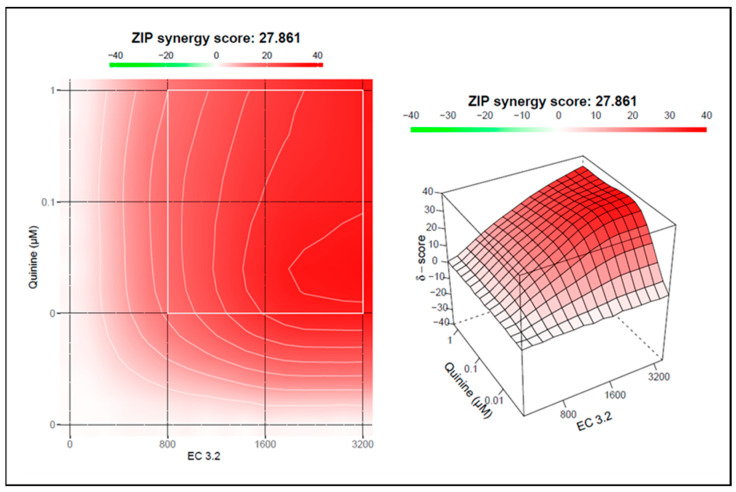
Interaction profile of quinine and EC 3.2 for determining the synergy in inhibiting the replication capacity of Wuhan-type SARS-CoV-2. Drug interactions were analyzed using the reference model Bliss independence. The illustrations were created using the open-source and free web application SynergyFinder. The synergy calculations were performed on data derived from the experiments in Calu-3 cells. The data represent means of three independent experiments. A color-coded interaction graphic was used to illustrate the Bliss synergy scores. High synergy scores are colored in red.

To analyze if the combinational treatment of EC 3.2 and quinine exhibits synergistic antiviral activity on Wuhan-type SARS-CoV-2, the Bliss independence model was used again. The conducted small molecule interaction analysis resulted in a Bliss synergy score of 27.8 for the combination of EC 3.2 and quinine for inhibiting replication of Wuhan-type SARS-CoV-2 (Figure 7).

In summary, the results clearly show that the combinatorial treatment of EC 3.2 and quinine, in addition to IAV (Figure 3 and Figure 4), exhibits a very strong synergistic effect in inhibiting replication of SARS-CoV-2.

**Figure 8:**
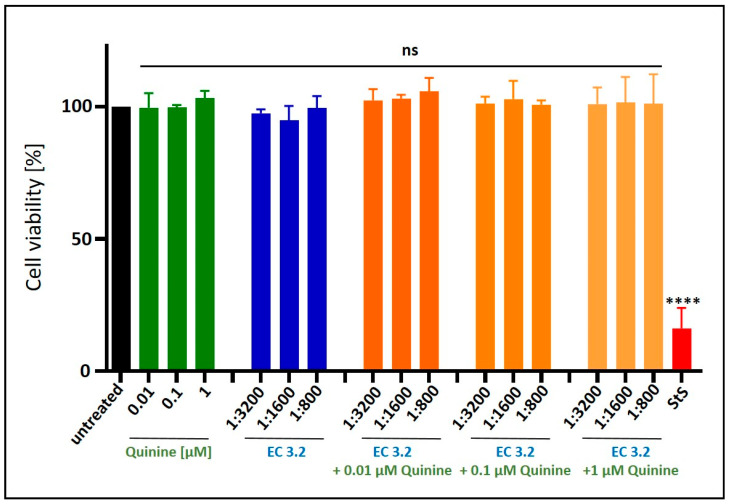
Influence of the combinatory treatment of quinine with EC 3.2 on the cell viability of Calu-3 cells**.** Following treatment with different dilutions of EC 3.2 or concentrations of quinine sulfate (indicated at the *x*-axis) for three days, the influence on cell viability was measured via water-soluble tetrazolium salt (WST)-1 assay. Bars represent means of three independent experiments ± SD. Staurosporine (StS, 1 µM) was used as a positive control. Statistical analysis was performed using a multiple comparison Kruskal–Wallis test (Anova) followed by Dunn’s post hoc test (**** *p* < 0.0001 and ns = not significant versus the untreated control).

To verify that the combined treatment of EC 3.2 and quinine did not exert nonspecific cytotoxic effects, WST-1 assays were performed on uninfected Calu-3 cells under the same conditions as the infection experiments. No loss of cell viability was observed for any of the tested dilutions or concentrations (Figure 8). Staurosporine (1 µM) served as a positive control.

### 3.4. Antiviral Activity of ElderCraft^®^ Q-Complex Against IAV and SARS-CoV-2 in Comparison to ElderCraft^®^ Without Quinine

We showed that an anthocyanin-rich elderberry fruit extract (EC 3.2) and quinine possess antiviral properties against IAV and SARS-CoV-2 (Figure 1) and that these agents act synergistically (Figure 3, Figure 4, Figure 6, and Figure 7). Thus, we aimed to analyze a commercially available product that integrates these two compounds into a single formulation, christened as “ElderCraft^®^ Q-Complex”. By evaluating ElderCraft^®^ Q-Complex, we aimed to determine its efficacy as a practical and accessible antiviral agent, potentially offering enhanced synergistic effects in the inhibition of viral replication.

**Figure 9:**
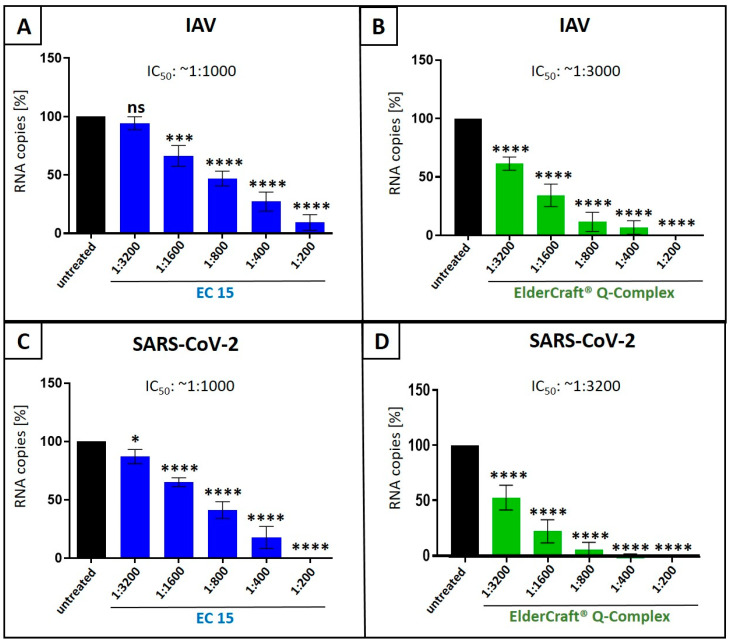
Antiviral activity of ElderCraft^®^ Q-Complex against IAV and SARS-CoV-2 compared to EC 15. (**A**,**B**) MDCKII cells were infected with the IAV isolate A/Puerto Rico/8/34 (PR8) at an MOI of 0.01. Thirty minutes after infection and removal of input virus, cells were treated with indicated dilution steps of EC 15 (**A**) or ElderCraft^®^ Q-Complex (**B**). Cell culture supernatants were harvested at 2 days post infection (dpi). The virions were purified and analyzed via qRT-PCR. Bars show mean values of three independent experiments ± standard deviation. Statistical analysis was performed using a multiple comparison Kruskal–Wallis test (Anova) followed by Dunn’s post hoc test (*** *p* < 0.0002; **** *p* < 0.0001; and ns = not significant versus the untreated control). (**C**,**D**) Calu-3 cells were infected with the clinical isolate SARS-CoV-2_PR-1_ at an MOI of 2 × 10^−2^. One hour after infection and removal of input virus, cells were treated with indicated concentrations of EC 15 (**C**) or ElderCraft^®^ Q-Complex (**D**). Cell culture supernatants were harvested at 3 dpi. The virions were purified and analyzed via qRT-PCR. Bars show mean values of four independent experiments ± standard deviation. Statistical analysis was performed using a multiple comparison Kruskal–Wallis test (Anova) followed by Dunn’s post hoc test (* *p* < 0.0192 and **** *p* < 0.0001 versus the untreated control).

Therefore, we compared the antiviral activity of ElderCraft^®^ Q-Complex with ElderCraft^®^ (EC 15, 15% anthocyanins) alone following infection of MDCKII cells with IAV_PR-8_ (Figure 9A,B) or Calu-3 cells with Wuhan-type SARS-CoV-2 (Figure 9C,D). ElderCraft^®^ Q-Complex contains similar amounts of anthocyanins as EC 15; however, in addition, quinine was added in an amount of 10 mg per 15 mg anthocyanins. The infection with IAV_PR-8_ or Wuhan-type SARS-CoV-2 was performed as described before (Figure 1, Figure 3, and Figure 6), and qRT-PCR analysis was conducted (Figure 9).

Following treatment with increasing amounts of EC 15 (1:3200–1:200), a dose-dependent reduction in the replication of IAV (Figure 9A) or SARS-CoV-2 (Figure 9C) was detectable, resulting in an IC_50_ of ~1:1000 for both IAV and SARS-CoV-2. Treatment with the identical dilution series of ElderCraft^®^ Q-Complex, where additional quinine was added, resulted in an IC_50_ of ~1:3000 for inhibiting replication of IAV (Figure 9B) and an IC_50_ ~1:3200 for SARS-CoV-2 (Figure 9D). Thus, it could be concluded that a combinational formulation of black elderberry fruit extract and quinine resulted in an about 3-fold better reduction in viral replication in comparison to the treatment with black elderberry fruit extract alone.

**Figure 10:**
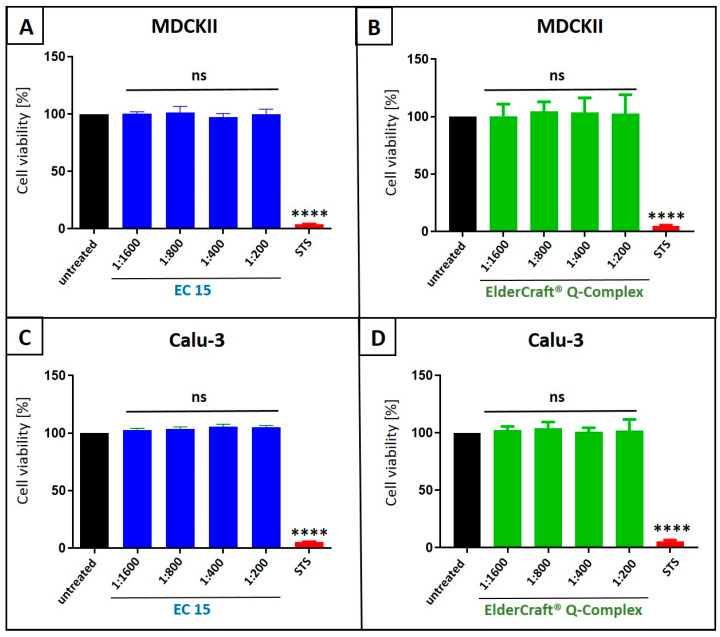
Influence of EC 15 (**A**,**C**) or ElderCraft^®^ Q-Complex (**B**,**D**) on the cell viability of MDCKII (**A**,**B**) and Calu-3 (**C**,**D**) cells. Following treatment with different dilutions of EC 15 (**A**,**C**) or ElderCraft^®^ Q-Complex (**B**,**D**) (indicated at the *x*-axis) for two (**A**,**B**) or three (**C**,**D**) days, the influence on cell viability was measured via water-soluble tetrazolium salt (WST)-1 assay. Bars represent means of three independent experiments ± SD. Staurosporine (StS, 1 µM) was used as a positive control. Statistical analysis was performed using a multiple comparison Kruskal–Wallis test (Anova) followed by Dunn’s post hoc test (**** *p* < 0.0001 and ns = not significant versus the untreated control).

To assess potential nonspecific effects of EC 15 or ElderCraft^®^ Q-Complex treatment on cell viability, WST-1 assays were performed on uninfected MDCKII (Figure 10A,B) and Calu-3 cells (Figure 10C,D) under conditions identical to the infection experiments. No cytotoxicity was observed at any tested dilution (Figure 10). Staurosporine (1 µM) served as a positive control.

## 4. Discussion

The continuous emergence of new variants of SARS-CoV-2 as well as IAV have been posing serious global health and socioeconomic challenges. Moreover, it remains highly likely that new viruses or virus variants capable of causing pandemic threats will emerge in the future. This was previously the case for MERS, SARS-CoV-2, and various historical IAV pandemics, including the Spanish, Asian, Hong Kong, and Russian flu viruses. In particular, RNA viruses such as corona- and orthomyxoviruses exhibit high mutation rates due to the error-prone viral RNA-dependent RNA polymerase or shifts in viral genome sequences, providing one reason for the extremely high evolutionary dynamic of both SARS-CoV-2 and IAV [37]. This situation underscores the urgent need for the development of new antiviral agents as part of a comprehensive pandemic preparedness strategy.

Effective, existing antiviral treatment options for SARS-CoV-2 as well as IAV infections encounter some remaining problems: They have to be administered early after infection and, moreover, development of drug resistance has been observed frequently, as well as side effects. Additionally, those drugs are expensive and, therefore, unavailable to a large part of the world’s population [38,39]. In the last years, antiviral activity in vitro and in vivo was reported for several natural products, and some of them entered clinical studies [38,40,41]. One example is iota-carrageenan, a high molecular weight sulfated polymer extracted from red seaweed. It was shown that iota-carrageenan exhibits antiviral activity against several respiratory viruses, among them IAV and SARS-CoV-2 [42,43,44,45,46,47]. Most importantly, during a randomized, placebo-controlled, double-blinded, multicenter clinical study, a relative risk reduction of 79.8% for SARS-CoV-2 infections was achieved when healthcare workers in a COVID-19 station used a nasal spray containing iota-carrageenan [48]. Iota-carrageenan-containing nose spray and lozenges are sold prescription free in more than 40 countries worldwide. Moreover, another clinical trial with a nasal spray containing iota-carrageenan and Ivermectin demonstrated a reduction in disease severity following SARS-CoV-2 infection [49]. Similar to carrageenans, most natural products are easily distributable and have significantly fewer side effects than chemically synthesized drugs. Moreover, they are broadly active and exhibit a low risk for the development of resistance, as they mostly inhibit host cell targets [38].

This study demonstrates that the combination of the two natural substances—European black elderberry fruit extract and quinine—exhibits a strong synergistic antiviral effect against SARS-CoV-2 and IAV in vitro without affecting cell viability. The results suggest that this combinational approach might represent an effective treatment option against various respiratory RNA viruses and variants thereof. To our knowledge, this is the first time that quinine and an elderberry fruit preparation have been combined for medicinal purposes, either in traditional practice or in formal research. This novel combination represents an innovative strategy, uniting two historically independent remedies to achieve synergistic antiviral effects.

Juices and different fruit extracts and have previously been demonstrated to represent potential sources for antiviral agents. Thereby, extracts from fruits such as blackberry, blackcurrant, mulberry, and pomegranate show antiviral activity against a variety of viruses. Among them are Dengue virus, SARS-CoV-2, Hepatitis C virus, Poliovirus, IAV, Zika virus, and Human immunodeficiency virus type 1 [50].

*S. nigra*, commonly known as European black elderberry, has been utilized in traditional medicine for centuries to alleviate symptoms associated with viral infections. Historically, its use has been particularly prevalent in managing upper respiratory infections [51]. Over the last three decades, the therapeutic potential of black elderberry has gained scientific validation through several clinical trials. These studies have consistently demonstrated that extracts from black elderberry fruits are effective in reducing both the duration and severity of upper respiratory infections [13,14,52]. In parallel with clinical findings, numerous in vitro studies have sought to elucidate the mechanisms underlying properties of black elderberry.

Three main hypotheses have emerged from this body of research: (i) specific components within black elderberry, such as flavonoids and phenolic acids, are believed to exert direct antiviral effects by interfering with the viral lifecycle [16,19,53,54]; (ii) another proposed mechanism involves the inhibition of viral enzymes critical for replication and proliferation, thereby impeding the virus’s ability to multiply within the host [18,55]; and (iii) polysaccharides present in black elderberry are thought to play a crucial role in stimulating the immune system, enhancing the body’s natural defense mechanisms against viral pathogens [56,57].

Preparations from European black elderberry fruits have demonstrated a strong safety profile in both clinical and regulatory evaluations. Toxicological assessments indicate that properly prepared elderberry products—free from cyanogenic glycosides found in raw plant parts—pose no significant cytotoxic risk [58]. Clinical trials have reported no severe adverse effects, with occasional mild gastrointestinal symptoms being the most common [13,14,52,59]. Overall, elderberry is well tolerated when used within established guidelines, reinforcing its role as a safe natural health product.

Quinine has been previously shown to exert antiviral activity against various viruses [16,24,25,26,27,28]. Mechanistically, mainly three different hypotheses for its antiviral activity have been discussed: (i) quinine was shown to be a weak base and, thus, is able to increase intracellular the pH of acidic organelles, e.g., endosomes or lysosomes [60]. This step is crucial for the receptor-mediated endocytosis of different viruses and could, therefore, be one explanation for the antiviral activity of quinine in the early stages of viral replication [21,61]. (ii) It was demonstrated that quinine enhances the synthesis and, thus, the release of IFN-α. The released IFN-α then binds to the IFN-α–receptor and, thereby, stimulates the 2-5 (A) synthetase that activates RNAse L. The RNAse L then degrades viral RNA and, thus, inhibits the replication of different RNA viruses, such as SARS-CoV-2 and IAV [21,62]. (iii) Quinine also exhibits immunoregulatory properties, which can contribute to its antiviral activity. It was shown that quinine causes a reduction in the inflammatory response by inhibiting the production of pro-inflammatory factors [21,63].

As quinine is one of the oldest used drugs, its pharmacokinetics have been well elaborated. Various forms of extracts of the bark of the Cinchona tree (indigenous to the Andes of South America) have been used for hundreds of years, almost worldwide [23]. The Cinchona bark contains up to 15% of two alkaloids, quinine and quinidine, which are stereoisomers of each other [23]. The first recorded medical application of quinine dates back to 1630, where the countess of Chinchon living in Peru was successfully treated from malaria with extracts of the bark of the so called “fever tree”, which was later termed as Cinchona bark, and finally christened as quinine. After pure isolation in 1820 and chemical synthesis in 1944, it became the unrivaled antimalarial drug available that ultimately permitted the deployment of a stable British population in malaria-infested tropical colonies [64]. Due to its anti-malaria activity, quinine was commercialized in the 19th century as the so-called “Indian Quinine Tonic”, the typical beverage in tropical areas. The tonic water originates from the British soldiers who mixed quinine with lime and gin to overcome the bitter taste of the bark extracts. Even Winston Churchill said “*The gin and tonic has saved more Englishmen′s lives, and minds, than all the doctors in the Empire*” [65].

Currently, quinine is used for the treatment of complicated malaria tropica, namely as a reserve drug in cases of resistance to other malaria therapeutics. The standard dosage for treatment is 10 mg/kg quinine sulfate every 8 h, corresponding to approx. 2 g per day [66]. The standard dosage for malaria prophylaxis is 300 mg quinine once daily [23]. In addition, quinine is used in the treatment of nightly calf cramps due to its muscle-relaxing properties, with 200 mg quinine sulfate taken twice daily [67]. Long-term intake of more than 2 g quinine per day can cause a complex of side effects and symptoms consisting of hearing and balance disorders (tinnitus, hearing loss, dizziness, visual disturbances) and central nervous system effects (headache, confusion, delirium), generally summarized as so-called “quinonismus”. In most cases, these symptoms are quickly reversible [21,68]. Toxic doses, which lead to respiratory paralysis, are described for an ingested amount over 8–10 g [21]. In the European Union, it is actually allowed to add quinine up to 100 mg/kg to food or up to 85 mg/L to beverages and up to 250 mg/L to alcoholic beverages [69]. For example, in 1 l of tonic water, 85 mg quinine are present, which leads to a plasma concentration of ~0.5 µg/mL, which is equal to a molarity of ~1.5 µM [70,71]. One tablet of quinine, which is approved for the treatment of calf cramps or malaria prophylaxes, contains 200 mg of quinine sulfate, which correlates to a plasma concentration of ~2.9 µM quinine.

However, our combinational experiments clearly show that ~100 nM quinine in combination with elderberry fruit extract is able to completely block the replication of SARS-CoV-2 or IAV in vitro (Figure 3 and Figure 6). Altogether, our in vitro studies indicate that antiviral effects of the combinatorial treatment could be achieved at concentrations of quinine that would be several times below the plasma values reached by consumption of quinine-containing beverages or the uptake of one standard quinine tablet. Therefore, no concern about adverse effects for continuous application of these two natural products can currently be expected. However, translating these findings into in vivo efficacy and safety requires further investigation.

A possible explanation for the strong synergetic antiviral activity of the combinational treatment of elderberry fruit extract and quinine (Figure 3, Figure 4, and Figure 9) is the fact that they target different steps of viral life cycle. Synergistic activity of chemically synthesized drugs has been shown previously for SARS-CoV-2 and IAV [72]. For instance, in the case of IAV, Favipiravir, a viral RNA polymerase inhibitor was tested in combination with Oseltamivir, a neuraminidase inhibitor. Thereby, it was shown that these two small molecules exhibit synergistic antiviral activity, most likely by acting at different stages of the IAV replication cycle [73]. For SARS-CoV-2, a promising synergistic antiviral activity was shown for the polymerase inhibitor Molnupiravir in combination with Tilorone, an interferon inductor [74]. However, synergistic activity using two natural products against IAV or SARS-CoV-2 has not been shown yet.

For black elderberry extract, it was demonstrated before that, e.g., flavonoids, which are present in significant amounts in the extract used in this study [16], are able to inhibit the entry of IAV by directly binding to the virus [19]. In addition, isoquercetin, another component of black elderberry extract, blocks the attachment of IAV at the cell [75]. Both of these mechanisms act at earlier stages of viral replication than quinine. On the other hand, in the case of SARS-CoV-2, it is unlikely that black elderberry extract acts at early stages of viral replication, as we showed previously that the extract has no influence on SARS-CoV-2 replication prior to infection [16]. Some reports revealed that anthocyanins or phenolic compounds present in black elderberry extract could inhibit later steps in viral replication. For instance, it was described that these compounds block the active pocket of the neuraminidase of IAV or the papain-like protease of SARS-CoV-2, and thus act during late stages of viral replication [18,76]. In addition to its antiviral activity, both natural products, black elderberry extract as well as quinine, were shown to modulate the immune system and, thereby, might have an influence on the spread of infection in vivo [21,56,57,63].

The combination of standardized elderberry fruit extract and quinine offers several additional advantages, in terms of cost and efficacy. Standardized extracts rich in anthocyanins and polyphenols, derived from fruits, are relatively expensive. Human studies indicate that a daily dose of 60 mg to 90 mg of anthocyanins is required for effective treatment and prevention, making these natural products costly and often unaffordable for the general population [13]. In general, elderberry fruit extract is well tolerated, even at high doses, with no known issues related to tolerability. However, the stability of elderberry extract in liquid formulations presents a challenge. Anthocyanins degrade over time in aqueous conditions, necessitating over-dosing by manufacturers to compensate for the degradation [77]. This practice further increases the cost per dose. While our data suggest that EC 3.2 is chemically stable under storage conditions and likely under the experimental conditions used, we acknowledge that anthocyanin degradation could occur to some extent, and this issue warrants further investigation in future studies.

In addition to elderberry fruit extract, similar antiviral activities have to be expected in extracts obtained from other plant components of *S. nigra*, like flower or bark extracts, which will be the subject of further studies.

The search for solutions to reduce the daily required dose of polyphenols has led to exploring the synergy between quinine and black elderberry fruit extract. Our findings demonstrate that the combination of quinine with elderberry extract can enhance the efficacy of the antiviral effect (Figure 3, Figure 4, and Figure 9). This synergy allows for a reduction in the required daily dose of polyphenols while maintaining, or even improving, the therapeutic benefits. This reduction not only makes the treatment more cost effective but also enhances its accessibility for a broader population. Additionally, by improving stability and reducing the need for over-dosing, the combination minimizes the economic burden on manufacturers and consumers alike.

The potential clinical application of EC 3.2 and quinine lies in their broad antiviral activity and synergy, which may allow for lower effective doses, reducing the risk of side effects. Given quinine’s established pharmacokinetics and elderberry’s historical use in managing respiratory infections, this combination could be repurposed as an adjunct therapy alongside existing antiviral treatments such as neuraminidase inhibitors for Influenza or protease inhibitors for SARS-CoV-2. Currently, there are no incompatibilities known for the usage of black elderberry extracts or quinine in addition to standard antiviral drugs, which would contradict our combinatorial approach in addition to antiviral treatment of IAV or SARS-CoV-2 infections. Nevertheless, future clinical trials should explore their efficacy in combination with standard treatments, assess pharmacodynamic interactions, and determine whether they can enhance patient outcomes or serve as an accessible alternative in resource-limited settings.

## 5. Conclusions

This study demonstrated that European black elderberry fruit extract and quinine inhibit the in vitro replication of the two major respiratory RNA viruses, SARS-CoV-2 and Influenza A. Most intriguingly, a combinational treatment synergistically enhances antiviral efficacy at lower individual concentrations of both natural substances without compromising cell viability. These findings suggest a promising, accessible therapeutic antiviral strategy and warrant further evaluation in vivo.

## 6. Patents

Iprona Lana SpA has filed a PCT and EP patent entitled “Combination of Elderberry Extract and Quinine for the Prevention and Treatment of RNA Virus Infections”, claiming the priority date of 31 August 2023.

## Data Availability

The data from each individual experiment carried out in this study are included in Supplementary Table S2.

## Supplementary Materials

The following supporting information can be downloaded at: https://www.mdpi.com/article/10.3390/nu17071205/s1, Figure S1. Anthocyanin stability at 4 °C in ElderCraft 3.2% measured by the pH Differential Method; Table S1. Composition of EC 3.2 and EC 15 used in this study. Table S2. Data of all individual experiments carried out in the study.
