# Supplementary material for: Synergistic Antiviral Activity of European Black Elderberry Fruit Extract and Quinine Against SARS-CoV-2 and Influenza A Virusa
Source: Nutrients. 2025 Mar 29;17(7):1205. doi: 10.3390/nu17071205 (PMC11990106; doi:10.3390/nu17071205)
Supplement: Supplementary file 1 [file nutrients-17-01205-s001.zip › Supplementary Figure S1.pptx]

## Slide 1
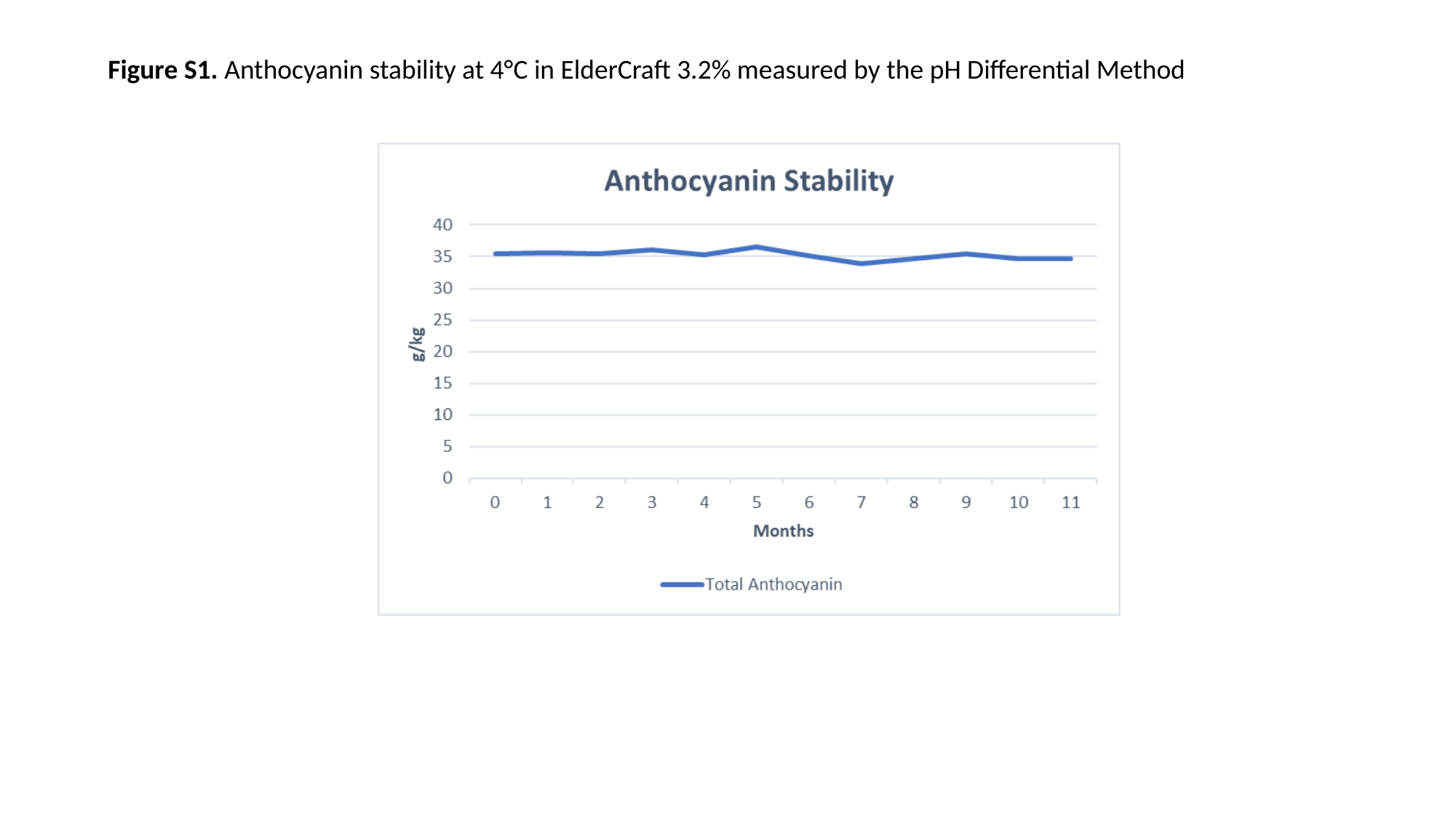

Figure S1. Anthocyanin stability at 4°C in ElderCraft 3.2% measured by the pH Differential Method
