# Supplementary material for: Synergistic Antiviral Activity of European Black Elderberry Fruit Extract and Quinine Against SARS-CoV-2 and Influenza A Virusa
Source: Nutrients. 2025 Mar 29;17(7):1205. doi: 10.3390/nu17071205 (PMC11990106; doi:10.3390/nu17071205)
Supplement: Supplementary file 1 [file nutrients-17-01205-s001.zip › Supplementary Table S1.pdf]

Supplementary Table S1. Composition of EC 3.2 and EC 15 used in this study.

EC 15	ElderCraft 15%, European Black Elderberry Extract (powder)	Batch 2401308733	
**Analysis**	**Method**	**Unit**	**Value**
Total Acid Content (Z, pH 8.1)	(anhydrous, IFU 3)	g / kg	151,63
Dry Matter	(Drying at 105°C)	%	96,29
Moisture	(Drying at 105°C)	%	3,71
Anthocyanins	as Cya-3-glu (spectrum through pH-Diff.)	g / kg	172,69
Polyphenols	as Catechin (Folin Ciocalteu)	g / kg	283,79
Polyphenols	(as Gallic Acid, Folin)	g / kg	248,48
Hydroxycinnamic Acid Derivatives	Chlorogenic Acid (HPLC)	mg / kg	6545,90
Hydroxycinnamic Acid Derivatives	Neochlorogenic Acid (HPLC)	mg / kg	257,00
Flavonol	Rutin (Quercetin-3-Rut.) (HPLC)	mg / kg	27949,20
Flavonol	Isoquercitrin (Quercetin-3-Glu) (HPLC)	mg / kg	2450,30
Flavonol	Kaempferol-3-Rut. (HPLC)	mg / kg	364,80
Flavonol	Isorhamnetin-3-Rut. (HPLC)	mg / kg	101,80
Flavonol	Total Flavonols	%	3,08

EC 3.2	ElderCraft 3,2%, European Black Elderberry Extract (liquid)	Batch 2201309506	
**Analysis**	**Method**	**Unit**	**Value**
pH Value	(IFU 11)		3,16
Total acidity (Z) pH 8.1	(anhydr., IFU 3)	g / kg	46,20
Anthocyanins	as Cya-3-glu (pH-Diff.)	g / kg	36,90
Polyphenols	as Catechin (Folin Ciocalteu)	g / kg	56,89
Polyphenols	as Gallic acid, (Folin Ciocalteu))	g / kg	51,31
Hydroxycinnamic acid derivatives	Chlorogenic acid (HPLC)	mg / kg	1336,00
Hydroxycinnamic acid derivatives	Neochlorogenic acid (HPLC)	mg / kg	54,00
Flavonols	Rutin (Quercetin-3-Rut. (HPLC)	mg / kg	7072,00
Flavonols	Isoquercitrin (Quercetin-3-Glu (HPLC)	mg / kg	553,00
Flavonols	Kaempferol-3-Rut. (HPLC)	mg / kg	74,00
Flavonols	Isorhamnetin-3-Rut. (HPLC)	mg / kg	31,00
Flavonols	Isorhamnetin-3-Glu. (HPLC)	mg / kg	6,00
