# Supplementary material for: Synergistic Antiviral Activity of European Black Elderberry Fruit Extract and Quinine Against SARS-CoV-2 and Influenza A Virusa
Source: Nutrients. 2025 Mar 29;17(7):1205. doi: 10.3390/nu17071205 (PMC11990106; doi:10.3390/nu17071205)
Supplement: Supplementary file 1 [file nutrients-17-01205-s001.zip › Supplementary Table S2.pdf]

Supplementary Table S2. Data of all individual experiments carried out in the study.

Figure 1

	Experiment 1		Experiment 2		Experiment 3	
	Quantity qRT-PCR	%	Quantity qRT-PCR	%	Quantity qRT-PCR	%
untreated	36359,88867	100	71569,21208	100	41996,90234	100
0.01 µM Quinine	35841,78566	98,5744122	65000,70833	90,82217	42563,95964	101,350236
0.1 µM Quinine	20962,08821	57,65233366	34609,68132	48,35834	27090,76693	64,50658362
1 µM Quinine	11454,71126	31,5037	15275,04411	21,34304	16067,99154	38,25994452
10 µM Quinine	4179,479899	11,49475	7382,814779	10,31563	7406,027018	17,63469829
untreated	36359,88867	100	71569,21208	100	41996,90234	100
1:800 EC 3.2	32609,54119	89,689	52468,72428	73,31187	36302,76042	86,44151923
1:400 EC 3.2	22037,82345	60,61026	31911,51595	44,58833	23197,44271	55,23608031
1:200 EC 3.2	8648,667318	23,78629	11197,95809	15,64633	10343,72656	24,62973692

Figure 2

	Experiment 1		Experiment 2		Experiment 3	
		cell viabilty %		cell viabilty %		cell viabilty %
untreated		100		100		100
0.01 µM Quinine		99,56079653		97,98194791		101,9252098
0.1 µM Quinine		95,97065083		98,56405716		101,6321543
1 µM Quinine		97,71071907		98,98128852		100,7471114
10 µM Quinine		97,11424716		99,99001875		94,01509764
100 µM Quinine		85,5786233		87,36063584		66,84513891
untreated		100		100		100
1:800 EC 3.2		104,2226825		99,96862404		107,5101127
1:400 EC 3.2		101,4537702		103,5261911		104,0733475
1:200 EC 3.2		99,33148495		100,3581549		101,6735693
1:100 EC 3.2		101,0824095		100,074331		98,31915695
1:50 EC 3.2		61,48943076		61,45382392		76,51534712

Figure 3

	Experiment 1		Experiment 2		Experiment 3	
	Quantity qRT-PCR	%	Quantity qRT-PCR	%	Quantity qRT-PCR	%
untreated	138671,8151	100	38551,73503	100	38363,21868	100
0.1 µM Quinine	114421,526	82,51246005	20000,95752	51,88082328	26070,32422	67,6242566
1 µM Quinine	73804,60156	53,22249623	8739,641439	22,66990431	11231,4117	29,1333495
1:800 EC 3.2	115135,4297	83,0272753	30209,62826	78,36126762	40504,72786	105,0659013
1:400 EC 3.2	73466,70052	52,97882664	19594,55762	50,82665567	20612,30534	53,46660877
1:200 EC 3.2	23973,1	17,28765141	12186,95443	31,61194799	7654,606812	19,85541457
0.1 + 1:800	67296,55469	48,52936744	10732,51863	27,83926228	19202,4209	49,80948558
0.1 + 1:400	29636,91146	21,37197918	5067,837545	13,14554985	3570,527832	9,261652763
0.1 + 1:200	0	0	1322,052124	3,42929345	946,4032389	2,454891429
1 + 1:800	6257,875	4,512723076	4046,276367	10,49570496	1534,482666	3,980320639
1 + 1:400	5700,589193	4,110849193	1929,53422	5,005051573	701,3740234	1,819305987
1 + 1:200	0	0	479,2877604	1,243232659	654,8024699	1,698503244

Figure 5

	Experiment 1		Experiment 2		Experiment 3	
		cell viability %		cell viability %		cell viability %
untreated		100		100		100
0.1 µM Quinine		100,08556		104,308735		103,16
1 µM Quinine		98,3785685		106,068329		96,162
1:800 EC 3.2		98,8134615		104,850086		96,84
1:400 EC 3.2		96,1723661		104,434491		100,874
1:200 EC 3.2		96,428436		105,360703		91,5925639
0.1 + 1:800		98,1916684		103,14689		103,901897
0.1 + 1:400		99,7663361		103,794299		98,9686015
0.1 + 1:200		98,393996		100,885925		101,785591
1 + 1:800		101,171898		100,19045		100,826483
1 + 1:400		98,0497002		100,78568		100,311532
1 + 1:200		96,4317255		101,212316		102,792263

Figure 6

	Experiment 1		Experiment 2		Experiment 3	
	Quantity qRT-PCR	%	Quantity qRT-PCR	%	Quantity qRT-PCR	%
untreated	5222001,5	100	2110596,719	100	6257739,25	100
0.01 µM Quinine	4815013,406	92,20628156	2009216	95,19658503	5048890,563	80,68234167
0.1 µM Quinine	3426733,188	65,62106862	1546347,328	73,26588326	3858806,625	61,66454802
1 µM Quinine	2263056,844	43,33696273	1119399,875	53,03712761	2563535,625	40,96584282
1:3200 EC 3.2	4332203,219	82,96058932	1517466,328	71,89750248	5508455,625	88,02628881
1:1600 EC 3.2	3140126,188	60,13261749	1407796,688	66,70135867	4301849,313	68,7444641
1:800 EC 3.2	1453605,156	27,83616888	939635,5938	44,51990214	2011472,594	32,14375853
0.01 + 1:3200	1691469,938	32,39121891	965307,4531	45,73623395	1588793,531	25,38925749
0.01 + 1:1600	950061,3125	18,19343239	630775,7969	29,88613558	996727,8438	15,92792227
0.01 + 1:800	564561,6563	10,81121207	358727,6719	16,99650477	616177,875	9,846653086
0.1 + 1:3200	982664,7813	18,81778052	592382,4375	28,06705953	669191,5938	10,69382355
0.1 + 1:1600	635020,4688	12,1604804	278746,9063	13,20701884	659738,4688	10,54276061
0.1 + 1:800	151793,9063	2,906814681	120778,9375	5,722501908	41900,25	0,669574879
1 + 1:3200	442556,75	8,474849155	210383,7813	9,967976325	147937,5313	2,364073116
1 + 1:1600	145949,125	2,794888607	55767,03125	2,642240024	69659,71875	1,113177075
1 + 1:800	73442,46875	1,406404589	15776,375	0,747484105	7742,875	0,123732784

**Figure 8**						
	**Experiment 1**		**Experiment 2**		**Experiment 3**	
		cell viability %		cell viability %		cell viability %
untreated		100		100		100
0.01 µM Quinine		98,77202791		94,7193231		105,623318
0.1 µM Quinine		99,374755		99,3624812		100,874451
1 µM Quinine		101,2576714		106,414736		102,442719
1:3200 EC 3.2		99,07505315		97,5453146		96,075
1:1600 EC 3.2		100,0821299		95,4503835		89,336
1:800 EC 3.2		99,49804346		104,124116		95,2390554
0.01 + 1:3200		98,69992863		101,535155		107,135
0.01 + 1:1600		104,7315618		102,61752		102,26592
0.01 + 1:800		103,562197		102,194328		111,632665
0.1 + 1:3200		100,3118035		99,4898047		104,194925
0.1 + 1:1600		99,04664154		99,0159247		110,863752
0.1 + 1:800		99,81699553		100,172952		102,601378
1 + 1:3200		96,90787548		98,0909413		108,292825
1 + 1:1600		95,52254662		97,0055475		112,716626
1 + 1:800		95,93623194		93,7493582		114,017316

Figure 9 A+B						
	**Experiment 1**		**Experiment 2**		**Experiment 3**	
	Quantity qRT-PCR	%	Quantity qRT-PCR	%	Quantity qRT-PCR	%
EC 15						
untreated	38992,28809	100	204573,8418	100	49778,54199	100
3200	35071,2207	89,94399258	189398,7715	92,58210621	50092,17529	100,6300572
1600	26567,59713	68,13551714	116243,4355	56,82223813	36956,82869	74,24248925
800	16831,81152	43,16702699	89096,31152	43,55215248	27006,11768	54,25252849
400	7019,76815	18,00296544	62384,10913	30,49466568	16615,74316	33,37932872
200	1943,213867	4,98358512	12571,41602	6,145172768	8519,357422	17,11451778
Q-Complex						
untreated	86799,64502	100	28691,58203	100	49778,54199	100
3200	53814,88965	61,99897435	15895,17896	55,40014816	33193,97852	66,68330808
1600	28529,88607	32,86866676	7267,609375	25,3301103	22143,34375	44,48371299
800	2668,610474	3,074448603	5547,335449	19,33436589	6204,607422	12,46442176
400	0	0	3039,728516	10,59449602	4778,05957	9,598633024
200	0	0	0	0	0	0

Figure 9 C+D								
	**Experiment 1**		**Experiment 2**		**Experiment 3**		**Experiment 4**	
	Quantity qRT-PCR	%	Quantity qRT-PCR	%	Quantity qRT-PCR	%	Quantity qRT-PCR	%
EC 15								
untreated	6231643	100	946153,4063	100	534883,1172	100	559933,5313	100
3200	5104082,438	81,90588642	794501,625	83,97175551	462810,0625	86,52545718	535693,6875	95,67094264
1600	3812393,125	61,17797706	626936,1146	66,26157137	339726,6797	63,51419007	391628,916	69,94203672
800	1984421,656	31,84427696	368531,9023	38,95054437	250277,0586	46,79098116	263371,0859	47,03613398
400	845914,96	13,57450932	58404,94027	6,172882736	133578,8539	24,97346609	145839,9124	26,04593301
200	0	0	0	0	0	0	0	0
Q-Complex								
untreated	3157166,75	100	825256,0118	100	563038,2204	100	1940549,165	100
3200	1150507,209	36,44112903	438201,3516	53,09883785	334039,1597	59,32797236	1185775,129	61,10513201
1600	196748,6973	6,231812028	230636,7108	27,94729242	143494,4019	25,48573022	542670,7656	27,96480375
800	1463,082566	0,046341631	10799,06277	1,308571233	30103,54868	5,346626141	285600,9934	14,71753453
400	163,0695602	0,00516506	231,5284405	0,028055347	411,7422638	0,073128653	37931,17017	1,954661642
200	0	0	0	0	2,897251129	0,000514575	0,691448033	3,56316E-05

Figure 10 A+B

	Experiment 1		Experiment 2		Experiment 3	
		cell viability %		cell viability %		cell viability %
EC 15						
untreated		100		100		100
1600		102,380118		99,3484331		99,3223034
800		102,085932		106,292495		96,0346482
400		94,2983918		100,842867		96,5689176
200		101,198162		103,58391		95,2789186
Q-Complex						
untreated		100		100		100
1600		89,182563		101,339559		110,390865
800		97,4954792		101,741729		113,925759
400		93,4632603		98,7939737		118,12397
200		87,9818507		98,3875743		120,769105

Figure 10 C+D

	Experiment 1		Experiment 2		Experiment 3	
		cell viability %		cell viability %		cell viability %
EC 15						
untreated		100		100		100
1600		104,331241		101,645186		101,820546
800		104,690812		104,76781		101,608952
400		106,047112		107,290739		102,89447
200		104,723349		106,69598		103,479167
Q-Complex						
untreated		100		100		100
1600		105,105966		98,9973138		103,277559
800		105,136405		98,0361964		108,813804
400		102,33848		96,4841179		103,34403
200		96,1846122		95,4		113,296005
